# Genetic Association and Altered Gene Expression of *CYBB* in Multiple Sclerosis Patients

**DOI:** 10.3390/biomedicines6040117

**Published:** 2018-12-18

**Authors:** Giulia Cardamone, Elvezia Maria Paraboschi, Giulia Soldà, Stefano Duga, Janna Saarela, Rosanna Asselta

**Affiliations:** 1 Department of Biomedical Sciences, Humanitas University, Via Rita Levi Montalcini 4, 20090 Pieve Emanuele, Milan, Italy; giulia.cardamone@st.hunimed.eu (G.C.); elvezia_maria.paraboschi@hunimed.eu (E.M.P.); giulia.solda@hunimed.eu (G.S.); stefano.duga@hunimed.eu (S.D.); 2 Humanitas Clinical and Research Center, Via Manzoni 56, 20089 Rozzano, Milan, Italy; 3 Institute for Molecular Medicine Finland, Helsinki Institute of Life Science (HiLIFE), University of Helsinki, 00290 Helsinki, Finland; janna.saarela@helsinki.fi

**Keywords:** multiple sclerosis, association study, reactive oxygen species, NADPH oxidase, *CYBB*

## Abstract

Multiple sclerosis (MS) is a chronic neurological disorder characterized by inflammation, demyelination, and axonal damage. Increased levels of reactive oxygen species (ROS), produced by macrophages and leading to oxidative stress, have been implicated as mediators of demyelination and axonal injury in both MS and experimental autoimmune encephalomyelitis, the murine model of the disease. On the other hand, reduced ROS levels can increase susceptibility to autoimmunity. In this work, we screened for association with MS 11 single nucleotide polymorphisms (SNPs) and two microsatellite markers in the five genes (*NCF1*, *NCF2*, *NCF4*, *CYBA*, and *CYBB*) of the nicotinamide adenine dinucleotide phosphate (NADPH) oxidase (NOX2) system, the enzymatic pathway producing ROS in the brain and neural tissues, in 347 Finnish patients with MS and 714 unaffected family members. This analysis showed suggestive association signals for *NCF1* and *CYBB* (lowest *p* = 0.038 and *p* = 0.013, respectively). Functional relevance for disease predisposition was further supported for the *CYBB* gene, by microarray analysis in CD4^+/−^ mononuclear cells of 21 individuals from five Finnish multiplex MS families, as well as by real-time RT-PCRs performed on RNA extracted from peripheral blood mononuclear cells of an Italian replication cohort of 21 MS cases and 21 controls. Our results showed a sex-specific differential expression of *CYBB*, suggesting that this gene, and more in general the NOX2 system, deserve to be further investigated for their possible role in MS.

## 1. Introduction

Multiple sclerosis (MS) (Online Mendelian Inheritance in Men, OMIM #126200) is a chronic disease of the central nervous system (CNS) characterized by multifocal inflammation, plaques of myelin degeneration, axonal damage, and by a high degree of individual variability in the severity and progression of symptoms [1,2,3]. Among neurological disorders of young adults, MS is the most common: in Europe, where a latitude-correlated distribution of prevalence and incidence rates can be observed, prevalence ranges from 70 to 100 per 100,000, whereas incidence varies between 2 and 4 per 100,000 person/year; the highest prevalence and incidence rates have been reported in Finland [4]. Here, incidence and prevalence reach the exceptional rate of 11.6 and 200 per 100,000 in the Southern Ostrobothnian region (western coast of Finland), reflecting several centuries of genetic drift in a small and isolated population [5].

In agreement with the multigenic character of MS, genome-wide linkage scans, genome-wide association studies (GWAS), as well as meta-analyses performed in large cohorts disclosed more than 200 non-HLA (human leukocyte antigen) single nucleotide polymorphisms (SNPs) associated with MS, each having a small effect size on MS predisposition [6,7,8,9,10]. Typically, the identified association signals point to genes belonging to innate and adaptive pathways [9,10]. The deep involvement of a dysregulated immune system in MS is also supported by the efficacy of drugs targeting T- and B-cell functions in the treatment of the disease, as well as by the numerous studies indicating CD4^+^ and CD8^+^ T cells as strong contributors to the pathogenic process [11,12,13,14].

Apart from immune-mediated mechanisms, a growing body of evidence indicates that oxidative stress (OS) may play a role in the etiology of MS [15,16,17]. Increased levels of oxygen-free radicals (collectively called reactive oxygen species, ROS), produced by macrophages and leading to OS, have been implicated as mediators of demyelination and axonal injury in both MS and experimental autoimmune encephalomyelitis, the murine model of the disease [18,19,20]. Moreover, ROS: (i) activate specific transcription factors, such as the nuclear transcription factor kappa B, which in turn upregulates the expression of genes associated with MS or its progression (e.g., tumor necrosis factor α) [21,22]; (ii) mediate the activity of matrix metalloproteinases, which are involved in T-cell activation and trafficking into the CNS and hence probably involved in some of the early pro-inflammatory events in MS [23,24,25]; (iii) are produced in increased amount by activated mononuclear cells of patients, resulting in oxygen damage to DNA, lipids, and proteins—these molecules are frequently found in active MS lesions and are associated with apoptotic oligodendrocytes and neurodegeneration in the brains of patients with MS [16,26,27,28].

Besides reactions involving the electron-transport chain of mitochondria or those related to the metabolism of amino acids and neurotransmitters, in the brain and neural tissue ROS are also produced by enzymatic pathways, such as xanthine oxidase, lipoxygenase, and cyclooxygenase, as well as by the nicotinamide adenine dinucleotide phosphate (NADPH) oxidase (NOX) systems [29,30]. Specifically, the NOX2 system is a complex that, in resting cells, is compartmentalized between cytosol and plasma membrane: the core enzyme is composed of three cytoplasmic polypeptides (p47phox, p67phox, and p40phox, encoded by *NCF1*, *NCF2*, and *NCF4* genes, respectively), and a membrane-bound flavo-hemo-cytochrome, b558, constituted by two components, the p22phox and gp91phox subunits (encoded by *CYBA* and *CYBB*, respectively) [29,30]. When the resting cell is exposed to stimuli (i.e., chemotactic reactants or particulate stimuli, such as bacteria and fungi), p47phox becomes heavily phosphorylated, inducing all cytoplasmic components to translocate to the membrane-bound cytochrome in order to form the active enzyme complex [29,30].

Given the proposed role of OS in MS, its contribution to the pathogenesis of the disease was here investigated by genetic association and expression analyses of the five genes coding for the main components of the NOX2 complex.

## 2. Results

### 2.1. NCF1 and CYBB Are Associated with MS in the Finnish Population

For investigating the potential role of OS genes in the pathogenesis of MS, we performed an association analysis on a Finnish cohort of 63 MS families (547 individuals) with two microsatellite markers and 11 SNPs mapping in or located close to the five genes coding for the main components of the NOX2 complex (the soluble factors *NCF1*, *NCF2*, and *NCF4*, and the membrane-bound redox core proteins of the complex, *CYBA* and *CYBB*) (see Table S1 in the Supplementary Materials). No significant deviation from Hardy-Weinberg equilibrium (HWE) and no Mendelian inheritance errors were observed for any of the markers included in this study. The overall average genotype call rate was 96.5%, and the accuracy was >99.5% according to duplicated genotyping (5%) of all samples.

Table 1 shows the results of the allelic association analysis for each of the 11 variants for the whole study cohort. Two polymorphisms, D7S1870 in *NCF1* and rs5963310 in *CYBB*, showed suggestive evidence of association with MS in the transmission/disequilibrium (TDT) test (*p* = 0.038 and *p* = 0.027 for *NCF1* and *CYBB*, respectively; not corrected for multiple testing). By using the more powerful approach based on the TDT statistic implemented with the parental discordance test (see the Materials and Methods section), we confirmed the association signal for the rs5963310 polymorphism in *CYBB* (*p* = 0.013), which confers a protective effect against MS (the minor allele A is untransmitted in MS cases). No significant allelic association between MS and the other NADPH variants could be detected (Table 1).

### 2.2. CYBB Is Differentially Expressed in MS Cases

To verify the possible functional relevance of NADPH genes for MS predisposition, we performed a genome-wide microarray-based gene expression analysis. We extracted total RNA from CD4^+^ and CD4^−^ samples isolated from peripheral blood mononuclear cells (PBMCs) of 21 individuals from five Finnish multiplex MS families. An adequate amount of high-quality total RNA was obtained from nine MS cases (four males, five females) and ten controls (four males, six females) for the CD4^−^ cells, and from nine cases (five males, four females) and 11 controls (six males, five females) for the CD4^+^ cells. All patients had a relapsing-remitting (RR) subtype, i.e., the most common form of MS (~80% of cases), which is characterized by periods of acute intensification of symptoms followed by phases of almost complete remission [31].

To screen for differential expression of the genes of interest in MS, we specifically searched for probe sets corresponding to *NCF1*, *NCF2*, *NCF4*, *CYBA*, and *CYBB* in the NetAffx annotation site (http://www.affymetrix.com/analysis/index.affx). The 11 identified probes were individually searched in the University of California Santa Cruz (UCSC) Genome Browser (hg19; Blat search) to identify the correct target gene (http://genome.ucsc.edu/cgi-bin/hgGateway). Probe sets recognizing only intronic sequences or mapping to several loci were excluded: in this way expression data were available for seven probes mapping in the five genes.

**Figure 1:**
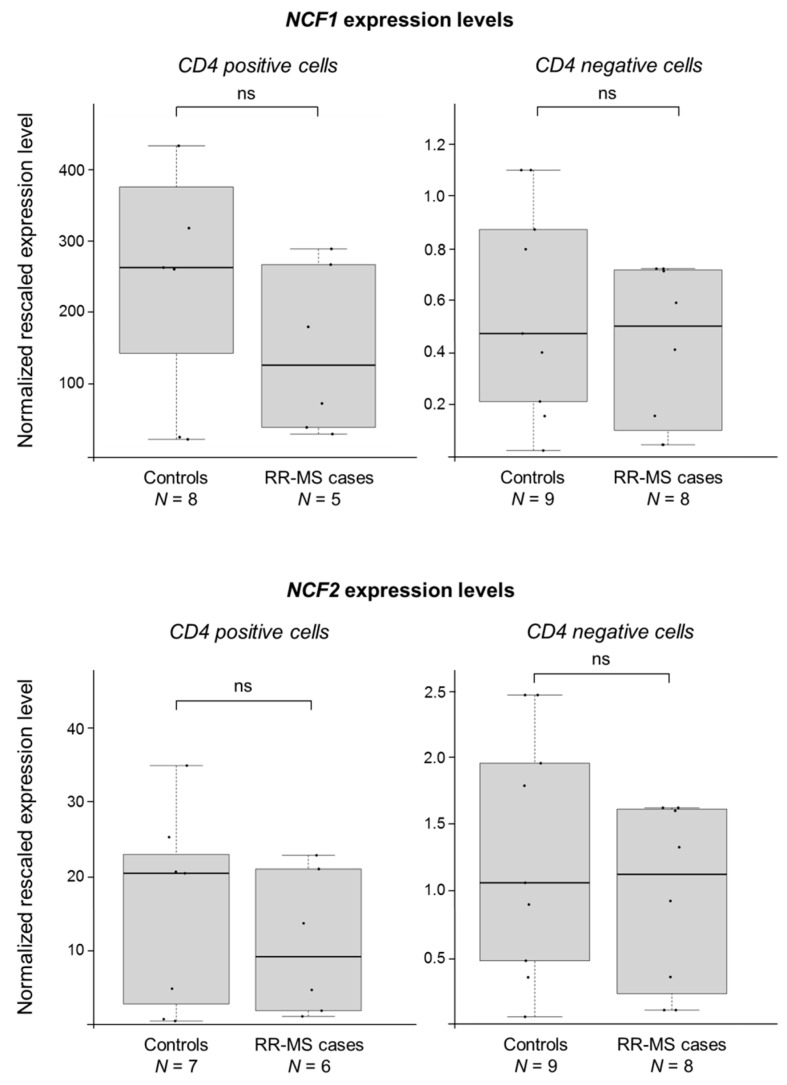
*NCF1* and *NCF2* do not show any significant difference between MS cases and controls. Boxplots show the expression levels of the *NCF1* and *NCF2* genes measured by semi-quantitative real-time RT-PCR in CD4^+^ and CD4^−^ cells (prepared from peripheral blood mononuclear cells, PBMCs). MS cases and controls belong to five multiplex Finnish families (comprising both male and female individuals). Boxes define the interquartile range; the thick line refers to the median. Results were normalized to expression levels of the *HMBS* and *ACTB* housekeeping genes and are presented as rescaled values. The number of subjects belonging to each group is indicated (*N*). The significance level of *t*-tests was in all cases not significant (ns).

As for the *NCF1* gene, none of the probes were mapping correctly/specifically. As for *NCF2*, it did not show appreciable expression signals in any of the CD4^+^/CD4^−^ samples. We hence monitored for potential differential expression of these two genes by using the more sensitive semi-quantitative real-time reverse transcription-PCR (RT-PCR) approach. Figure 1 shows the results of RT-PCR assays: no significant difference between cases and controls in the expression levels of either genes was evidenced (no significant difference between cases and controls was seen even stratifying individuals on the basis of the D7S1870 genotype in *NCF1*; data not shown).

**Figure 2:**
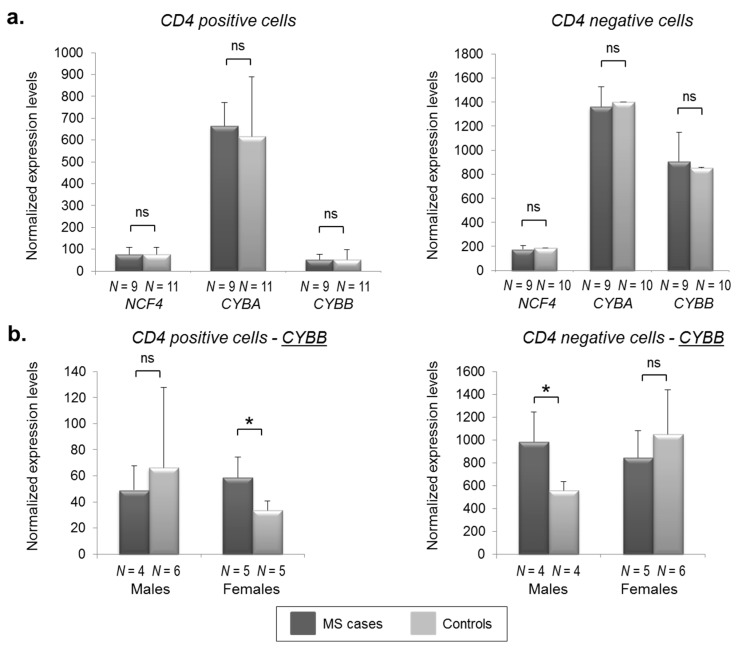
*CYBB* shows sex-related and cell-specific differential expression in MS cases and controls. *NCF4*, *CYBA*, and *CYBB* expression levels (shown by histograms) were measured by means of microarray-based experiments (see the “Materials and Methods” section). The number of subjects belonging to each group is indicated (*N*). Error bars represent means + SD (standard deviation). Significance levels of *t*-tests are shown. *: *p* < 0.05.

Concerning *NCF4*, *CYBA*, and *CYBB*, probes were correctly mapping on the corresponding transcripts and gave appreciable signals. In Figure 2a, differential expression analysis for MS cases and controls is shown for both CD4^−^ and CD4^+^ cells, again indicating no differences between the two groups. However, considering that the *CYBB* gene is located on chromosome X, we stratified patients according to gender. Interestingly, this analysis allowed us to highlight an opposite molecular signature not only between males and females, but also between CD4^−^ and CD4^+^ cells (Figure 2b). In particular, we found a 1.76-fold significant up-regulation (*p* = 0.021) of *CYBB* in RR-MS male patients in CD4^−^ cells (with females showing an opposite, though not significant, trend; 0.80-fold decrease). Conversely, in CD4^+^ cells, we noticed the opposite situation, with a 1.75-fold significant *CYBB* increased expression in RR-MS females patients (*p* = 0.014) and a 0.74-fold decrease (not significant) in MS male cases compared to healthy controls.

**Figure 3:**
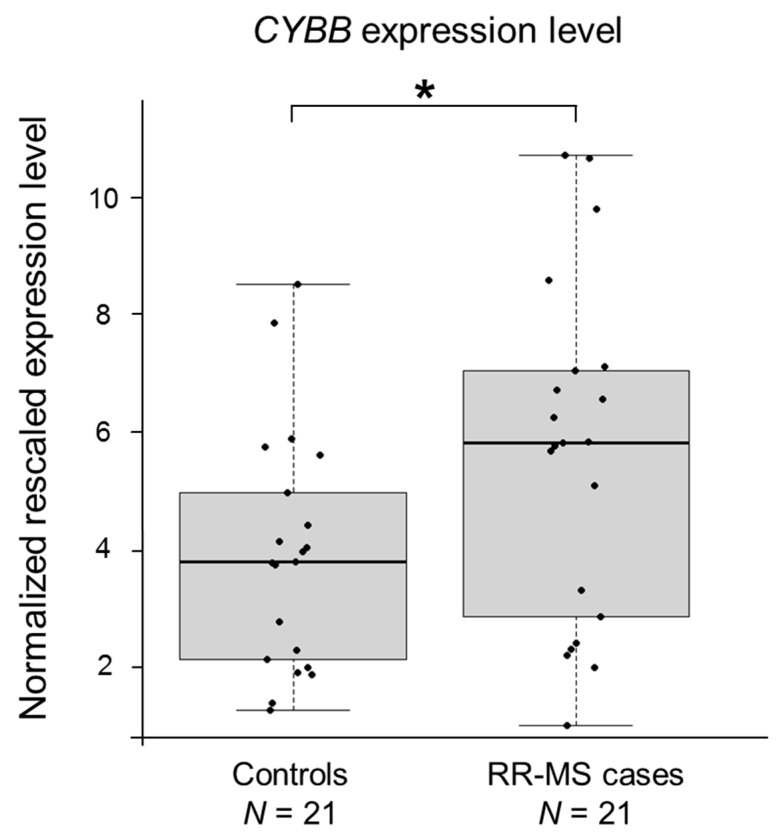
*CYBB* is upregulated in MS patients. Boxplots show the expression levels of the *CYBB* gene measured by semi-quantitative real-time RT-PCR in PBMCs of an Italian case-control cohort (only female individuals). Boxes define the interquartile range; the thick line refers to the median. Results were normalized to expression levels of the *HMBS* and *ACTB* housekeeping genes and are presented as rescaled values. The number of subjects belonging to each group is indicated (*N*). Significance levels of *t*-tests are shown. * *p* < 0.05.

To validate the most interesting result obtained from the microarray profiling, expression of *CYBB* was quantitated by real-time RT-PCR assays, performed on RNA extracted from PBMCs of an independent cohort of 21 RR-MS Italian cases and an equal number of age-matched controls. Only females were included in this analysis. We found a 1.43-fold significant up-regulation (*p* = 0.032) of *CYBB* in RR-MS patients (Figure 3), which is consistent with the high abundance of CD4^+^ cells in the heterogeneous pool of PBMCs (25–60% of PBMC is composed of CD4^+^ cells) [32].

## 3. Discussion

Apart from the well-known implication of mutations affecting the NOX2 complex in the pathogenesis of chronic granulomatous disease (CGD) [33], some information is currently available in the literature on the possible predisposing role of polymorphisms in *NCF1*, *NCF2*, *NCF4*, *CYBA*, and *CYBB* genes in other immune disorders. Among others, missense variants in the *NCF1* gene have been associated with systemic lupus erythematosus (SLE), Sjögren’s syndrome, and rheumatoid arthritis (RA) [34]; two missense polymorphisms in *NCF2* were described as predisposing to SLE [35]; and common polymorphisms in *NCF4* were associated both with RA and Crohn’s disease [36,37]. In our exploratory study, we aimed at investigating the possible genetic association between MS and all of the five genes coding for the NADPH oxidase complex. We found suggestive association signals for the *NCF1* and *CYBB* genes (lowest *p* = 0.038 and *p* = 0.013, respectively), which were paralleled, in the case of *CYBB*, by a gender-specific differential gene expression between MS cases and controls.

However, we acknowledge the limited size of our study material, and a lack of a replication step confirming the association of *CYBB* with MS. No significant association between the *CYBB* locus and MS was reported in the previous GWAS analyses, which, however, excluded the X chromosome harboring the *CYBB* gene from the genome-wide analysis. The role of the X chromosome in MS—a pathology characterized by higher susceptibility in females than males—remains still largely unknown. For instance, in the largest MS-related meta-analysis so far published, genome-wide, a total of 233 loci were found to be significantly associated with the disease, and only one maps to chromosome X [10]. The case-control analysis did not take gender into account, while the study presented here was utilizing family-based transmission analysis. Overall, the lack of association signals on chromosome X is indeed a common feature of complex traits: the X chromosome accounts for 5% of the nuclear genome and underlies almost 10% of Mendelian disorders [38]. Nonetheless, only 114 associations (0.8%) at *p* ≤ 5 × 10^−8^ have been reported on the X chromosome, on a total of more than 14,700 signals identified by GWAS for ~300 traits [39]. These data could potentially be explained by the fact that the vast majority of GWAS have not included chromosome X in their analyses and have not considered gender. There is also a lack of specific bioinformatics pipelines to adopt in the analytic steps [40].

To overcome the limitation of the lack of a replication cohort in the association study, we focused on the investigation of an “intermediate phenotype”, i.e., the gene expression profile of NOX2 components in CD4^+^ and CD4^−^ cells. We used a two-tiered approach, based on both microarray and real-time RT-PCR experiments. The most striking result was the differential gene expression for the *CYBB* gene in MS cases, with opposite molecular signatures not only between males and females, but also between CD4^−^ and CD4^+^ cells. Indeed, sex-differential expression has recently emerged as a common feature of many genes. For instance, by comprehensively analyzing the Genotype-Tissue Expression (GTEx) data across 53 tissues (publicly available at https://www.gtexportal.org/home/), Gershoni and Pietrokovski were able to demonstrate that more than 6500 protein-coding genes showed significant sex-differential expression in one or more tissues [41]. In most of the cases, these genes are differentially expressed according to gender in just one or a few tissues. The *CYBB* gene seems to conform to this rule, showing a certain degree of sex-differential expression in kidney cortex, bladder, and thyroid (see Figure S1 in the Supplementary Materials); similar observations can be made for *NCF1*, *NCF2*, *NCF4*, and *CYBA*. Of note, data relative to blood cell sub-populations are missing in the GTEx portal.

The differential gene expression evidenced by stratifying patients and controls on the basis of the gender and, above all, according to the cell type, could contribute to explaining—if confirmed in other immune disorders—some of the puzzling observations that have accumulated over the years on CGD, SLE, or RA. For instance, RA patients show in blood a marked increase in ROS formation, protein oxidation, as well as lipid peroxidation [42]. In addition, they also present a synovial tissue that is invaded by inflammatory cells, a large proportion of which are activated CD4^+^ T cells [43]. Both these phenomena can collectively account for tissue damage and for the chronicity of the disease. However, the development of RA has been associated with a lower copy number of the *NCF1* gene [44], and this association was supported by *ncf1* mutant rodents [45,46]. In this regard, it could be speculated that lower expression levels of the NOX2 complex could be protective against the chronic inflammation in RA relevant tissues; on the other hand, a lower oxidative burst response in antigen-presenting cells, especially in the thymus during priming, could change the antigen-presentation capacity of the cells, thus inducing autoimmunity [46]. Besides RA and SLE, previously shown examples of immune pathology characterized by inappropriate over-activation or down-regulation of ROS also include psoriasis, Hashimoto thyroiditis, vitiligo, and inflammatory bowel disease [47,48].

Concerning specifically MS, our results well fit with the observations of Fischer and colleagues [16], who described a global NOX2 over-expression in microglia and infiltrating macrophages of MS patients’ autopsy brain tissues. These data also suggest that an inflammation-associated oxidative burst could play a fundamental role in the demyelination process typical of MS.

In conclusion, our work adds another piece of information on the possible involvement of NOX2 in the pathogenesis of MS, suggesting that this particular topic deserves to be further investigated, especially in the light of potential therapies based on decreasing/enhancing the oxidative burst.

## 4. Materials and Methods

### 4.1. Materials

All oligonucleotides for SNP genotyping and quantitative real-time RT-PCRs were purchased from Proligo (Paris, France). Primer couples for microsatellite analysis were from Applied Biosystems (Foster City, CA, USA). All sequences can be provided on request. The AmpliTaq Gold, DynaZyme, and HotStar Taq DNA polymerases were from Applied Biosystems, Finnzymes (Espoo, Finland), and Qiagen (Hilden, Germany), respectively.

### 4.2. MS Pedigrees

The study was approved by the ethical committee of the Helsinki and Uusimaa hospital district (Decision 46/2002, #192/E9/02), and all individuals included in the study gave their informed consent. The study material consisted of a total of 63 families: 22 of them were multiplex MS families, having two to six affected cases per pedigree, whereas the remaining 41 families were composed of MS patients with their parents and unaffected siblings. These families were previously described [49] and they all originate from the high-risk region for MS in Southern Ostrobothnia, Finland. In total, the cohort consisted of 547 individuals, 116 being MS cases. Patient selection was hospital-based; only definite cases were included (clinically or laboratory-supported definite). Diagnosis of MS in affected individuals strictly followed Poser’s diagnostic criteria [50].

### 4.3. Genotyping

Genomic DNA was extracted from peripheral blood cells following standard procedures.

Two non-chimeric microsatellite markers (D7S1870 and D7S2518) were selected from databases for the association analysis of the *NCF1* gene, avoiding the highly duplicated regions characterizing this chromosomal locus (7q11.23). Microsatellite markers were PCR amplified in two-plex format in 10-μL reaction mixtures under standard conditions, using VIC (D7S1870) or PET (D7S2518) labeled primers, the AmpliTaq Gold DNA polymerase, a MJ Tetrad thermal cycler (MJ Research INC, Waltham, MA, USA), and a touch-down thermal profile. PCR products were run on an ABI-3730 Genetic Analyzer (Applied Biosystems) and analyzed using the Genotyper software (Applied Biosystems).

For the association analysis of the other genes, we selected 21 SNPs, having heterozygosity >10%. Primers flanking the putative SNPs were designed for multiplex PCR amplification using the web-based program MPprimer (https://omictools.com/mpprimer-tool?t=tab-tool-variant-0). SNPs were first tested for polymorphic content by direct sequencing the relevant PCR-amplified fragment in 32 healthy Finnish individuals. Nine SNPs were monomorphic in the Finnish population, whereas one was tri-allelic; those ten SNPs were not further investigated. The remaining 11 SNPs were typed in MS samples using an in-house developed microarray, based on allele-specific primer extension [51]. In particular, two allele-specific detection oligonucleotides were designed for each SNP: these primers were characterized by the presence of a 5’ aminolinker and a stretch of 9T (poly-T), followed by the SNP-specific sequence. The 5’ aminolinker and the poly-T served to spot and anchor detection oligonucleotides onto silane/isothiocyanate-coated chrome mirror microscope slides (Evaporated Coatings, Willow Grove, PA, USA). Spotting was performed in duplicate using an OmniGrid 100 arrayer (Discovery Scientific, Vancouver, BC, Canada). Regions containing the SNPs were PCR amplified with a touch-down protocol from genomic DNA in two multiplex reactions, using SNP-specific primers with a T3 (forward) or a T7 (reverse) tail. Multiplex reactions were in-vitro transcribed using the AmpliScribe T7 or T3 High Yield Transcription Kit (Epicentre Technologies, Madison, WI, USA). The DNAseI-treated T3 or T7 RNA pools were hybridized to the arrays in a hybridization buffer (1.67 M NaCl) at 42 °C for 20 min. Arrays were washed twice in a washing buffer (0.3 M NaCl, 0.5× TE, 0.1% TritonX-100) and subsequently rinsed in ice-cold water. The allele-specific extension was performed using the MMLV Reverse Transcriptase (Epicentre Technologies) in a 20-μL reaction containing 50 mM Tris-HCl (pH 8.3), 10 mM MgCl_2_, 75 mM KCl, 10 mM DTT, 0.5 μM dATP, 0.5 μM dGTP, 0.5 μM ddATP, 0.5 μM ddGTP, 1 μM Cy5-labeled dCTP, 1 μM Cy5-labeled dUTP (Cy5-labeled nucleotides were from Amersham Pharmacia Biotech, Uppsala, Sweden), 15% glycerol, and 0.24 M trehalose. Reverse transcription was carried out at 52 °C for 20 min. Finally, arrays were put in washing buffer under mild agitation for 15 min, dipped quickly in 50 mM NaOH, washed again in washing buffer, rinsed in ice-cold water, air dried, and scanned using a ScanArray 4000 instrument (Packard Biochip Technologies, Perkin Elmer Life Sciences, Boston, MA, USA). The image was analyzed using the ScanArray software (Packard Biochip Technologies, Perkin Elmer Life Sciences). Genotypes were called using an in-house developed software (W. Wong and C. Li, unpublished data).

The list of selected polymorphisms is presented in the Supplementary Materials (Table S1).

Important note: the entire analysis was performed a while ago, i.e., before the genomic databases became available with all the associated information on allele frequencies in different ethnicities.

### 4.4. Statistical Analyses

All genotypes were checked for Mendelian errors using PedCheck [52]. The check for the HWE was performed using the Genepop program (option 1), which also admits multiallelic markers [53]; only genotypes of women were considered for X-linked SNPs.

TDT [54] and HRR (a modified TDT test that uses siblings as “pseudo-controls”) [55] analyses, both implemented in the ANALYZE package [55], were used in the association study. Since the inheritance pattern of MS is unknown, analyses were performed using two different modes of inheritance, i.e., dominant with reduced penetrance of *f* = 0.05 (with disease allele-frequency estimate of 0.01) or *f* = 0.76 (with disease allele-frequency estimate of 0.0006) [56].

Analyses were repeated using the PLINK software v.1.07 [57] by applying a more powerful method based on the TDT statistic implemented with the parental discordance test. This test is based on counting the number of alleles in affected versus unaffected parents (using each nuclear-family parental pair as a matched pair). These counts are then combined with the transmitted and untransmitted counts of the basic TDT to give a combined test statistic [58]. In this analysis, multiallelic markers were analyzed as biallelic, considering from one hand the most common allele, and from the other all the remaining ones clumped together as they were a unique alternative allele. In the text, *p* values, ORs, and 95% CIs always referred to the minor allele.

Considering the exploratory hypothesis-generating nature of the present study and the limited sample size as compared to the number of investigated genetic variants, we did not adjust for multiple testing, and *p* values < 0.05 were considered to be statistically significant. However, the total number of performed tests as well as the threshold of significance based on the over-conservative Bonferroni correction has been indicated in the footnote of the relevant table.

### 4.5. RNA Samples

Fresh blood samples from 25 members (both male and female subjects) belonging to five of the above-mentioned multiplex MS families were collected. PBMCs were separated using a Ficoll-Paque (Amersham Pharmacia Biotech) density gradient centrifugation, and hence stratified to CD4^+^ and CD4^−^ cell populations by negative selection using the CD4^+^ T-cell isolation kit (Miltenyi Biotec, Auburn, CA, USA) and an autoMACS instrument (Miltenyi Biotec). Total RNA was extracted from CD4^+^ and CD4^−^ samples using the Trizol reagent (Ambion, Austin, TX, USA), treated with DNAse-I, and further purified with RNeasy Mini Kit columns (Qiagen, Valencia, CA, USA) according to the manufacturers’ instruction. RNA quality was assessed using the RNA 6000 Nano assay in a Bioanalyzer (Agilent Technologies, Foster City, CA, USA).

As for the replication cohort, we collected PBMCs from 21 unrelated RR-MS patients and 21 healthy subjects, all females and Caucasians (coming from Northern Italy). Controls were age-matched with cases. To avoid possible confounding effects, we focused only on RR-MS cases in remitting phase, who had not received any immune-modulatory therapy within the month prior to blood withdrawal. PBMCs were isolated from heparinized blood immediately after phlebotomy by centrifugation on a Lympholyte Cell separation media (Cederlane Laboratories Limited, Hornby, ON, Canada) gradient. Total RNA was isolated using the Eurozol kit (Euroclone, Wetherby, UK).

### 4.6. Sample Preparation, Microarray Processing, and Data Analysis

RNA samples were prepared for hybridization on the Affymetrix GeneChip Human Genome U133 Plus 2.0 Array (Affymetrix, Santa Clara, CA, USA) according to the manufacturer’s recommendations. In brief, 1 μg of total RNA was converted to biotin-labelled cRNA using the Affymetrix HT One-Cycle cDNA Synthesis Kit and the HT IVT Labelling Kit. Fifteen μg of cRNA were then fragmented and hybridized for 16 hours at 45 °C, washed in Affymetrix Fluidics Station 450, and then scanned with Affymetrix GeneChip Scanner 3000. Hybridization, washing, staining, and scanning were conducted using the same instruments for all samples.

Raw intensity data files were imported to GeneSpring 7.3 (Agilent Technologies) and GC Robust Multi-array Average (GC-RMA) normalized. We then excluded probe sets with low signal intensity (GC-RMA normalized signal < 50). Probe sets with a *p* ≤ 0.05 were considered to be differentially expressed.

### 4.7. Semi-Quantitative Real-Time RT-PCRs

Random hexamers and the TaqMan Gold RT-PCR kit (Applied Biosystems) were used to perform first-strand cDNA synthesis starting from 1 μg of total RNA from CD4^+/−^ cells, according to the manufacturer’s instructions. From a total of 20 µL of the RT reaction, 4 µL were used as template for semi-quantitative real-time RT-PCRs for the quantitation of the *NCF1* and *NCF2* mRNAs. Assays were carried out using the SYBR-Green Kit (Applied Biosystems) and a standard PCR thermal protocol on the ABI Prism 7900 HT Sequence Detection System. Data were analyzed with the Sequence Detector version 2.0 software (Applied Biosystems).

As for *CYBB* evaluation, random hexamers and the Superscript-III Reverse Transcriptase (Invitrogen, Carlsbad, CA, USA) were used in the RT step starting from 1 µg of RNA extracted from PBMCs of MS patients and controls. From a total of 20 µL of the RT reaction, 1 µL was used as a template for amplifications, using the FastStart SYBR Green Master Mix (Roche, Basel, Switzerland) on a LightCycler 480 (Roche), following a touch-down thermal protocol. Data were analyzed by the GeNorm software [59].

In all cases, RT-PCRs were performed at least in triplicate. Expression levels were normalized using *HMBS* (hydroxymethylbilane synthase gene) and *ACTB* (β-actin) as housekeeping genes. Primer sequences can be provided on request.

## Figures and Tables

**Table 1:** NADPH polymorphisms: Results of family-based association TDT and haplotype relative risk (HRR) tests.

Gene	Marker	ANALYZE Analyses ^1^		PLINK Analyses						
		HRR *p* Value	TDT *p* Value	Minor Allele ^2^	MAF	Transmitted/Untransmitted ^3^	TDT *p* Value	OR [95% CI]	*p* Par ^4^	*p* Comb ^4^
*NCF1*	D7S1870	**0.0491**	**0.0383**	20 repeats	0.4533	Untransmitted	0.330	0.727 [0.38–1.385]	0.157	0.206
	D7S2518	0.376	0.431	7 repeats	0.3986	/	1	1 [0.351–2.851]	0.414	0.655
*NCF2*	rs796860	0.895	0.500	C	0.06548	/	1	1 [0.250–3.998]	1	1
	rs2296164	0.568	0.399	T	0.4861	Untransmitted	0.297	0.643 [0.278–1.485]	0.564	0.239
	rs3818364	0.604	0.500	T	0.4688	Transmitted	0.847	1.0770 [0.506–2.291]	0.564	0.715
	rs789192	0.183	0.253	G	0.4268	Transmitted	0.144	1.727 [0.822–3.630]	0.317	0.423
	rs2274065	0.293	0.500	C	0.0974	Transmitted	0.180	4 [0.447–35.790]	0.317	0.414
*NCF4*	rs1883112	0.991	0.500	A	0.3861	Transmitted	0.353	1.417 [0.677–2.966]	0.257	0.182
	rs741999	0.680	0.444	A	0.4444	Transmitted	0.336	1.455 [0.675–3.134]	0.479	0.237
*CYBA*	rs4673	0.319	0.199	T	0.1795	Untransmitted	0.317	0.600 [0.218–1.651]	0.564	0.251
	rs2306422	0.661	0.500	T	0.494	Transmitted	0.739	1.118 [0.581–2.150]	0.655	0.876
*CYBB*	rs5963310	0.239	**0.0274**	A	0.09848	Untransmitted	0.0578	0.250 [0.0531–1.177]	0.0833	**0.0126**
	rs9330580	0.663	0.206	A	0.1203	Untransmitted	0.366	0.571 [0.167–1.952]	0.0833	0.109

Suggestive *p* values (*p* < 0.05) are indicated in bold. Results are presented not corrected for multiple testing (total number of performed analyses for each test: 11; Bonferroni threshold for significance: *p* < 0.0045). ^1^ Analyses were run both considering MS frequency of 0.01 and penetrance of 0.05, as well as considering MS frequency of 0.0006 and penetrance of 0.76 (identical results). ^2^ In the case of multiallelic markers (D7S1870, D7S2518), the minor allele corresponds to the most frequent repeat, which was analyzed against all the others clumped together. ^3^ Indication referred to the minor allele. ^4^ *p* par (*p* values for parents) and *p* comb (combined *p* value, deriving from the asymptotic TDT *p* value and the *p* par value). NADPH, nicotinamide adenine dinucleotide phosphate; CI, confidence interval; HRR, haplotype relative risk; MAF, minor allele frequency; OR, odds ratio; TDT, transmission disequilibrium test.

## Supplementary Materials

Supplementary materials can be found at http://www.mdpi.com/2227-9059/6/4/117/s1.

## Abbreviations

*ACTB*	β-actin
CD4^+^	Cluster of differentiation 4 positive
CD4^−^	Cluster of differentiation 4 negative
CGD	Chronic granulomatous disease
CI	Confidence interval
CNS	Central nervous system
*CYBA*	Cytochrome B-245 alpha chain, coding for the p22phox subunit of the NOX2 system
*CYBB*	Cytochrome B-245 beta chain, coding for the gp91phox subunit of the NOX2 system
GC-RMA	GC Robust Multi-array Average
GWAS	Genome-wide association study
HLA	Human leukocyte antigen
*HMBS*	Hydroxymethylbilane synthase gene
HRR	Haplotype relative risk
HWE	Hardy-Weinberg equilibrium
MMLV	Moloney Murine Leukemia Virus
MS	Multiple sclerosis
NADPH	nicotinamide adenine dinucleotide phosphate
*NCF1*	Neutrophil cytosolic factor 1, coding for the p47phox subunit of the NOX2 system
*NCF2*	Neutrophil cytosolic factor 2, coding for the p67phox subunit of the NOX2 system
*NCF4*	Neutrophil cytosolic factor 4, coding for the p40phox subunit of the NOX2 system
NOX	NADPH oxidase system
NOX2	NADPH oxidase system 2 (isoform 2 of the complex)
OMIM	Online Mendelian Inheritance in Men
OR	Odds ratio
OS	Oxidative stress
PBMCs	Peripheral blood mononuclear cells
RA	Rheumatoid arthritis
ROS	Reactive oxygen species
RR	Relapsing-remitting
RT-PCR	Reverse-transcription polymerase chain reactions
SD	Standard deviation
SLE	Systemic lupus erythematosus
SNP	Single nucleotide polymorphism
TDT	Transmission/disequilibrium test
