# Supplementary material for: Genetic Association and Altered Gene Expression of CYBB in Multiple Sclerosis Patients
Source: Biomedicines. 2018 Dec 18;6(4):117. doi: 10.3390/biomedicines6040117 (PMC6315774; doi:10.3390/biomedicines6040117)
Supplement: Supplementary file 1 [file biomedicines-06-00117-s001.pdf]

Article

Genetic association and altered gene expression of *CYBB* in Multiple Sclerosis patients

Giulia Cardamone [1], Elvezia Maria Paraboschi [1], Giulia Soldà [1,2], Stefano Duga [1,2], Janna Saarela [3], and Rosanna Asselta [1,2*]

[1] Department of Biomedical Sciences, Humanitas University, Via Rita Levi Montalcini 4, 20090 Pieve Emanuele, Milan, Italy; E-Mails: rosanna.asselta@hunimed.eu (R.A.); giulia.cardamone@st.hunimed.eu (G.C.); elvezia_maria.paraboschi@hunimed.eu (E.M.P.); giulia.solda@hunimed.eu (G.S.); stefano.duga@hunimed.eu (S.D.)

[2] Humanitas Clinical and Research Center, Via Manzoni 56, 20089 Rozzano, Milan, Italy; E-Mails: rosanna.asselta@hunimed.eu (R.A.); giulia.solda@hunimed.eu (G.S.); stefano.duga@hunimed.eu (S.D.)

[3] Institute for Molecular Medicine Finland, Helsinki Institute of Life Science (HiLIFE), University of Helsinki, Helsinki, 00290, Finland; E-Mail: janna.saarela@helsinki.fi (J.S.);

* Correspondence: rosanna.asselta@hunimed.eu; Tel: +39-02-82245215

Content:

Supplementary Table 1. NADPH polymorphisms selected for the association study.
Supplementary Figure 1. Sex-differential expression of *CYBB*.

Table S1. NADPH polymorphisms selected for the association study.

Gene (Chr)	*Protein* [1]	*Polymorphism*	*Function*	*RefSeq*	*Position* [2]
NCF1 (Chr7)	p47-phox	CA repeat	microsatellite mapping 61 kb from the TSS	AFMA060XC9 (D7S1870)	74,126,556 - 74,126,902
		CA repeat	microsatellite mapping 1,190 kb 3′ to *NCF1*	AFMC019YG9 (D7S2518)	75,377,845 - 75,378,207
NCF2 (Chr1)	p67-phox	A>C	3′ UTR variant	rs796860	183,524,855
		G>T	Intron 14	rs699244	183,525,452
		T>C	Intron 9	rs2296164	183,534,935
		T>G	Intron 4	rs3818364	183,542,747
		A>G	Intron 1	rs789192	183,556,291
		A>C	5′ UTR variant	rs2274065	183,559,704
NCF4 (Chr22)	p40-phox	G>A	-184 nt from TSS	rs1883112	37,256,846
		G>A	Intron 1	rs741999	37,259,273
		G>A	Intron 2	rs909484	37,260,601
		G>A	Intron 5	rs2072706	37,266,979
		C>T	Intron 8 [3]	rs2075939	37,271,882
		C>T	Exon 10, silent	rs1858	37,273,805
CYBA (Chr16)	p22-phox	G>A	3′ UTR	rs1049255	88,709,737
		C>T	Exon 6, Ala>Val	rs1049254	88,709,828
		C>T	Exon 4, His>Tyr	rs4673	88,713,236
		C>T	Intron 1	rs2306422	88,714,559
		G>A	5′ UTR	rs8854	88,718,865
		G>A	4.51 kb from the TSS (intronic in the *MVD* gene)	rs3736112	88,722,012
CYBB (ChrX)	gp91-phox	G>C	Intron 5	rs3935493	37,654,360
		T>A	Intron 7	rs5963310	37,659,562
		G>A	7 kb 3′ to *CYBB*	rs9330580	37,679,803

Chr, chromosome; TSS, transcription start site; UTR, untranslated region; nt, nucleotide; minus sign indicates the number of nucleotides of the promoter region upstream of the main TSS.

[1] UniProtKB/Swiss-Prot nomenclature is used for protein names.

[2] Chromosomal position (bp) according to UCSC Genome Browser on Human Feb. 2009 (GRCh37/hg19) assembly.

[3] The rs2075939 polymorphism can also determine a Pro>Leu missense change in the case of the transcript variant NM_013416.3 of the *NCF4* gene.

All polymorphisms were first tested for their polymorphic content by direct sequencing the relevant fragment in 32 healthy Finnish individuals; those resulted monomorphic are evidenced in gray, whereas the one showing three alleles (and hence not compatible with the genotype platform we used for genotyping) is evidenced in gray and it is underlined. These SNPs were not further analyzed.

In the examined population, the D7S1870 microsatellite showed alleles ranging from 12 to 25 CA repeats, whereas the D7S2518 marker from 3 to 14 CA repeats.

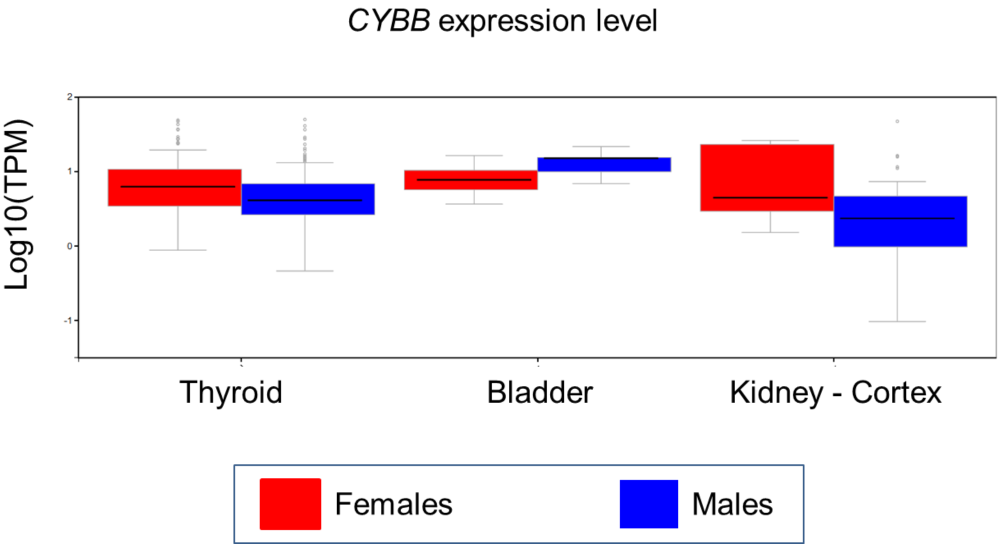

Figure S1. Sex-differential expression of *CYBB*. Data were retrieved from the GTEx repository (https://www.gtexportal.org/home/). Boxplots show the expression levels of the *CYBB* gene measured by RNAseq experiments in thyroid (153 females, 293 males), bladder (5 females, 6 males), and kidney cortex (9 females, 36 males). Expression values are shown in TPM (Transcripts per Million), calculated for a gene model with different isoforms collapsed to a single gene. No other normalization steps were applied. Box plots are shown with the median and 25% and 75% percentiles; points are displayed as outliers if above or below 1.5 times the interquartile range.
